# Supplementary material for: Demographic responses of a threatened, low-density ungulate to annual variation in meteorological and phenological conditions
Source: PLoS One. 2021 Oct 8;16(10):e0258136. doi: 10.1371/journal.pone.0258136 (PMC8500449; doi:10.1371/journal.pone.0258136)
Supplement: S1 Appendix — (DOCX) [file pone.0258136.s001.docx]

# S1 Appendix: Caribou Demographic Data

To assess the demographic response of woodland caribou to annual meteorological and phenological changes, we used data from 21 populations (or herds) situated in western Canada. These data included adult female survival, calf-to-adult female ratios and population growth rate. As noted in the main text, the number of monitoring years varied among populations and by demographic rate. Here, we show yearly estimates of calf-to-adult female ratios (Fig. S1.1) and adult female survival (Fig. S1.2) for each population. We also provide a graphical summary of the correlations among the three demographic rates and each rate’s distribution (Fig. S1.3).


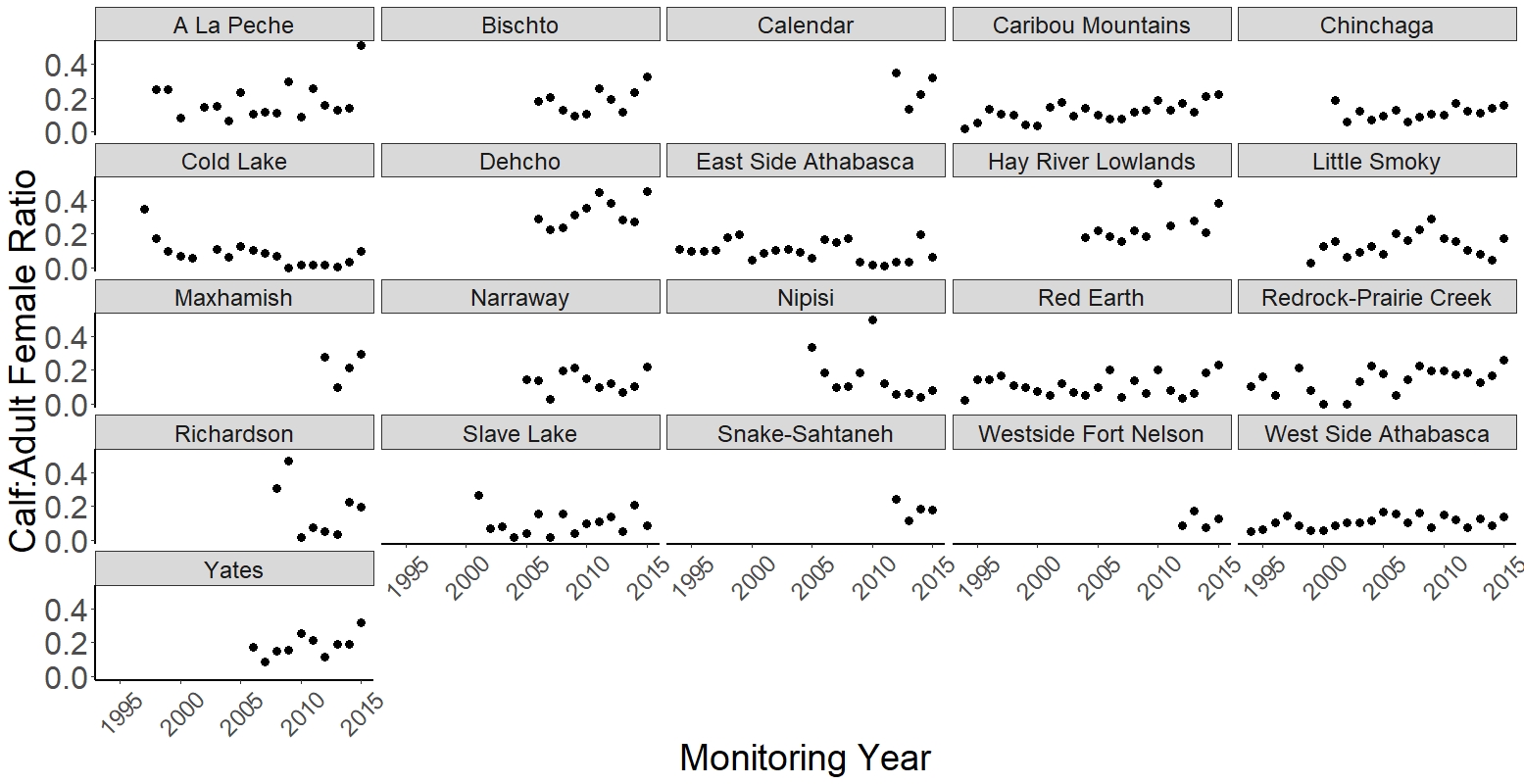


Figure S1.1: Annual calf-to-adult female ratios estimated for 21 herds of woodland caribou monitored for various intervals from 1995–2015. Data used to estimate ratios were collected during aerial surveys conducted during March when calves were ~ 9–10 months old.


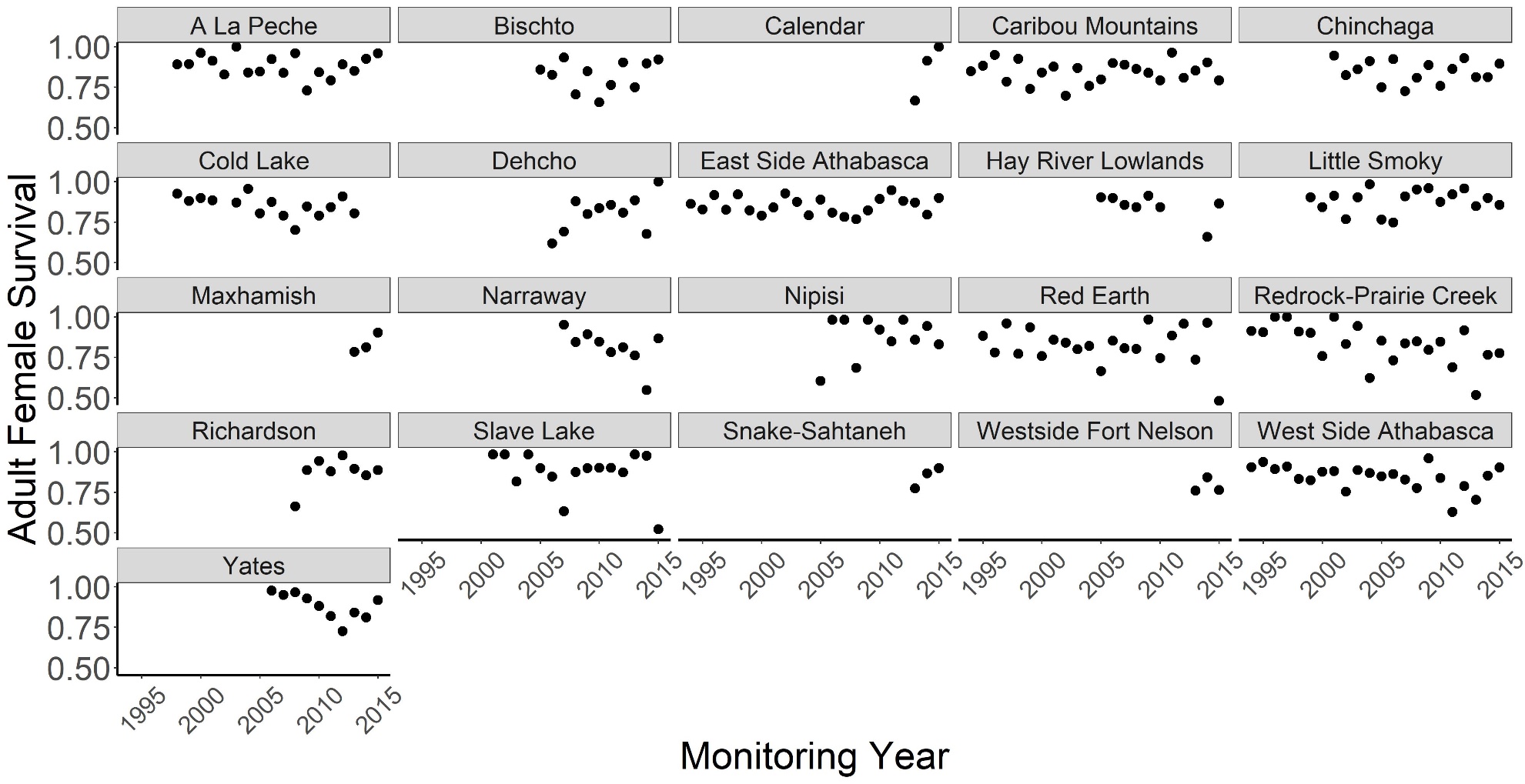


Figure S1.2: Annual estimates of adult female survival estimated for 21 herds of woodland caribou monitored for various intervals from 1995–2015. Estimates were derived using data from radio-collared females in each herd.


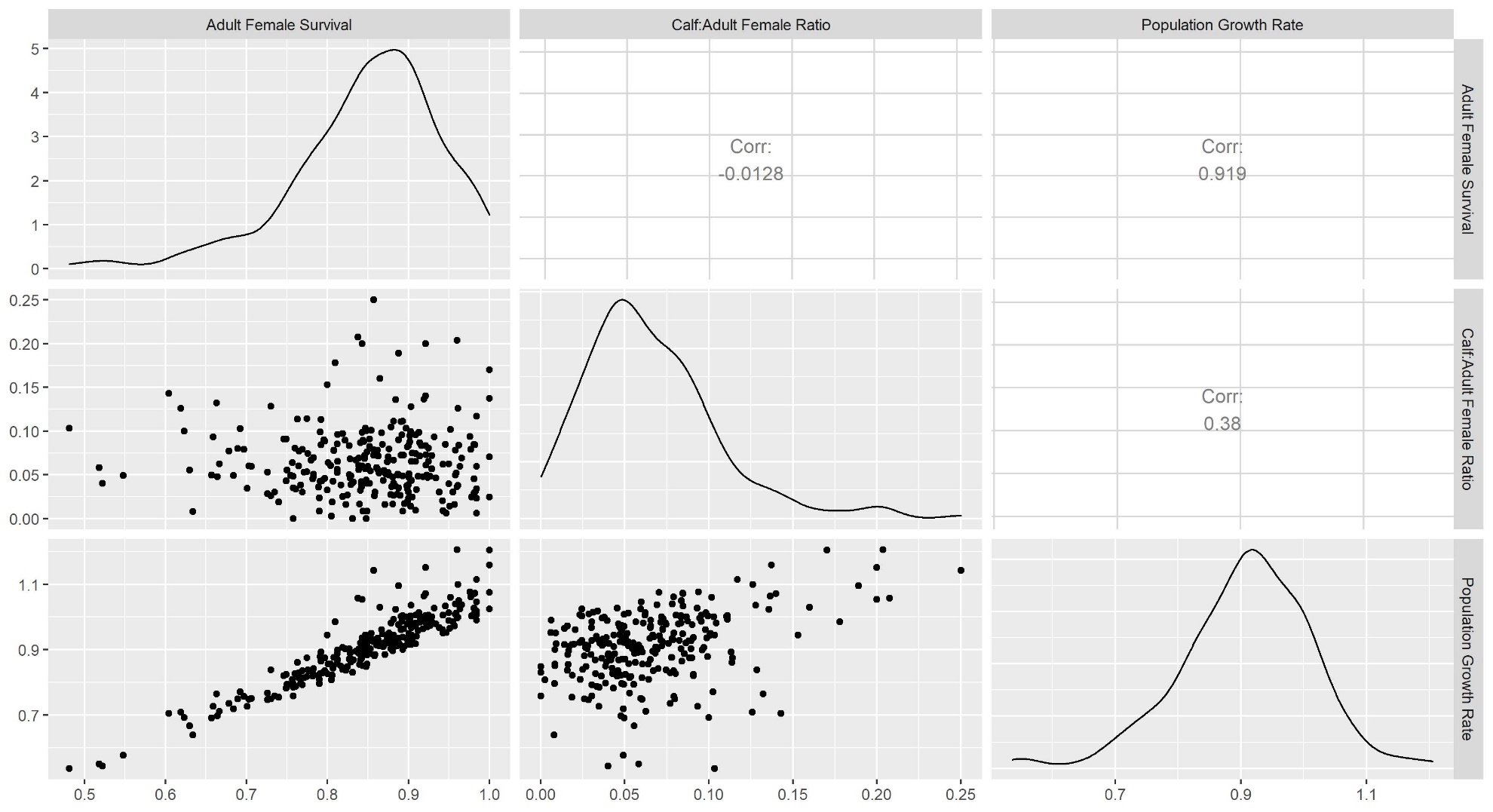


Figure S1.3: Relationships among adult female survival, calf: adult female ratios and population growth rate for 21 populations of woodland caribou. “Corr” indicates the strength of the correlation (measured by Pearson’s correlation coefficient) between the column and row variables. Line graphs on the diagonal show the probability density function of each demographic rate. Note that the x- and y-axes vary among graph facets.
