# Supplementary material for: Demographic responses of a threatened, low-density ungulate to annual variation in meteorological and phenological conditions
Source: PLoS One. 2021 Oct 8;16(10):e0258136. doi: 10.1371/journal.pone.0258136 (PMC8500449; doi:10.1371/journal.pone.0258136)
Supplement: S2 Appendix — (DOCX) [file pone.0258136.s002.docx]

**S2 Appendix: Delineating Seasons**

We assessed the demographic response of woodland caribou populations to climate-related variation within three uniquely defined seasonal periods. Two of these periods—the meteorological growing season and the snow season—were delineated using Daymet daily surface weather data available from the National Aeronautics and Space Administration’s Oak Ridge National Laboratory (1). The third period used Normalized Difference Vegetation Index (NDVI) data to delineate the growing season based on changes in plant phenology (see main text for NDVI data sources). Here, we provide further details on the methods used to delineate each seasonal period.

## Meteorological Growing Season

We defined the meteorological growing season as the annual period when the daily minimum temperature—averaged across a population’s range—consistently exceeded 0°C (i.e., the period between the last spring freeze and the first fall freeze). This threshold was relaxed to -3°C for the three caribou population situated in montane regions. We identified the start and end points of the meteorological growing season following the methods of Liu et al. (2). First, we calculated daily mean minimum temperatures for each population’s range using the Daymet data. We then fit a fifth-order polynomial equation to each population’s daily temperature data to smooth out day-to-day fluctuations. This smoothing reduced subjectivity in identifying the start and end of the growing season. We plotted the predictions of this smoothed curve against Julian day and delineated the meteorological growing season as the period whe*n* the expected mean minimal temperature exceeded 0°C (Fig. S2.1).


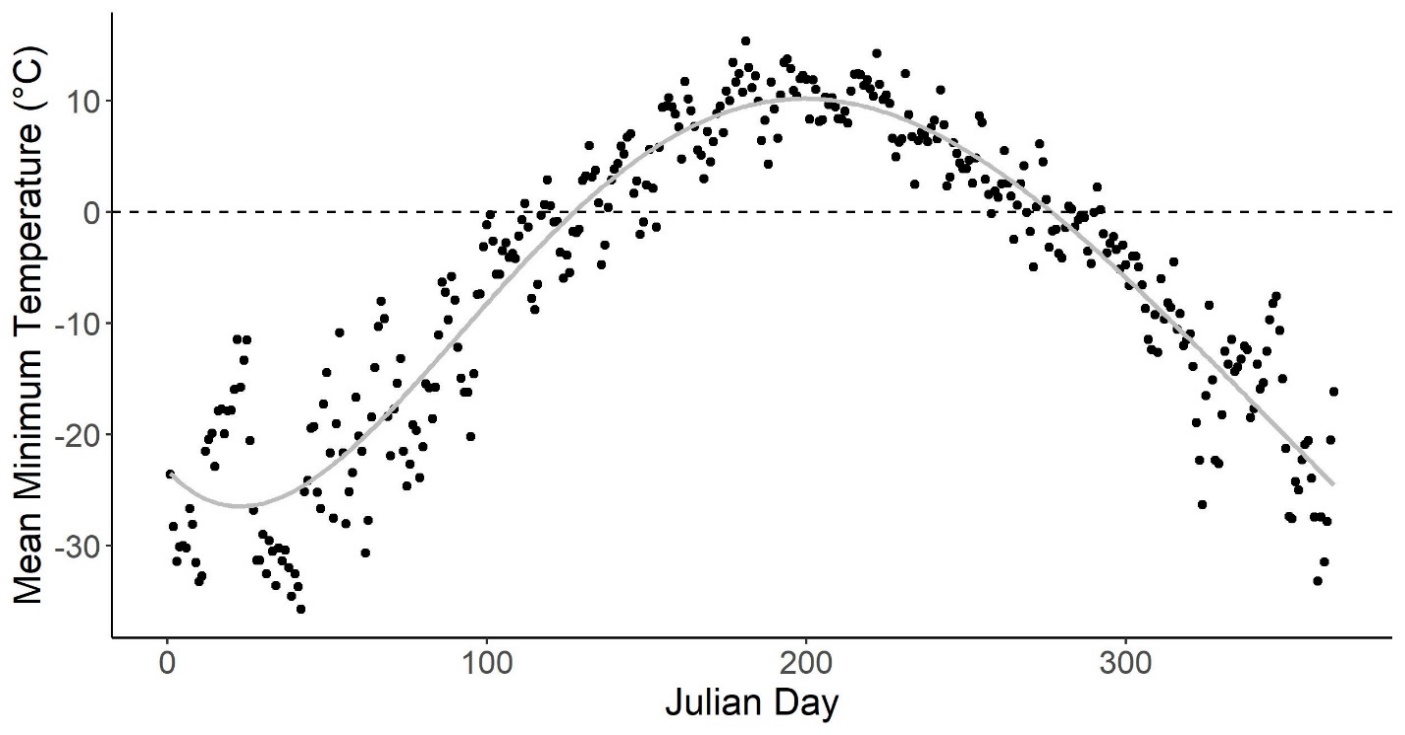
Figure S2.1: Daily changes in mean minimal temperature (°C) within the range of the Yates population of woodland caribou during 2015. The grey line represents a fifth-order polynomial equation fit to the observed values. The meteorological growing season was defined as the period whe*n* the expected mean minimal temperature exceeded 0°C.

## Phenological Growing Season

We used a similar approach for delineating the phenological growing season. NDVI data were derived from 16-day composite imagery and had a 250-m spatial resolution. For each 16-day time-step, we calculated a mean NDVI value for each caribou range. We then used the ‘modelNDVI’ function in the R package ‘phenex’ (3) to fit a smoothed curve to the mean NDVI values for each population-year. Prior to curve fitting, we specified the ‘best index slope extraction’ method with a sliding period of 20 to reduce noise in the data (4). These corrected-NDVI values were then smoothed by applying the commonly used Savitzky-Golay filter (5), retaining the default window size of 7 and polynomial degree of 2. After curve fitting (Fig. S2.2), we used the ‘phenoPhase’ function in the ’phenex’ package to identify greenup (the start of the growing season), senescence (the end of the growing season), the highest modelled NDVI value and the date when this value was reached. Greenup and senescence were identified using the default threshold of 0.55, which is the difference percentile between the maximum and minimum modelled NDVI values.


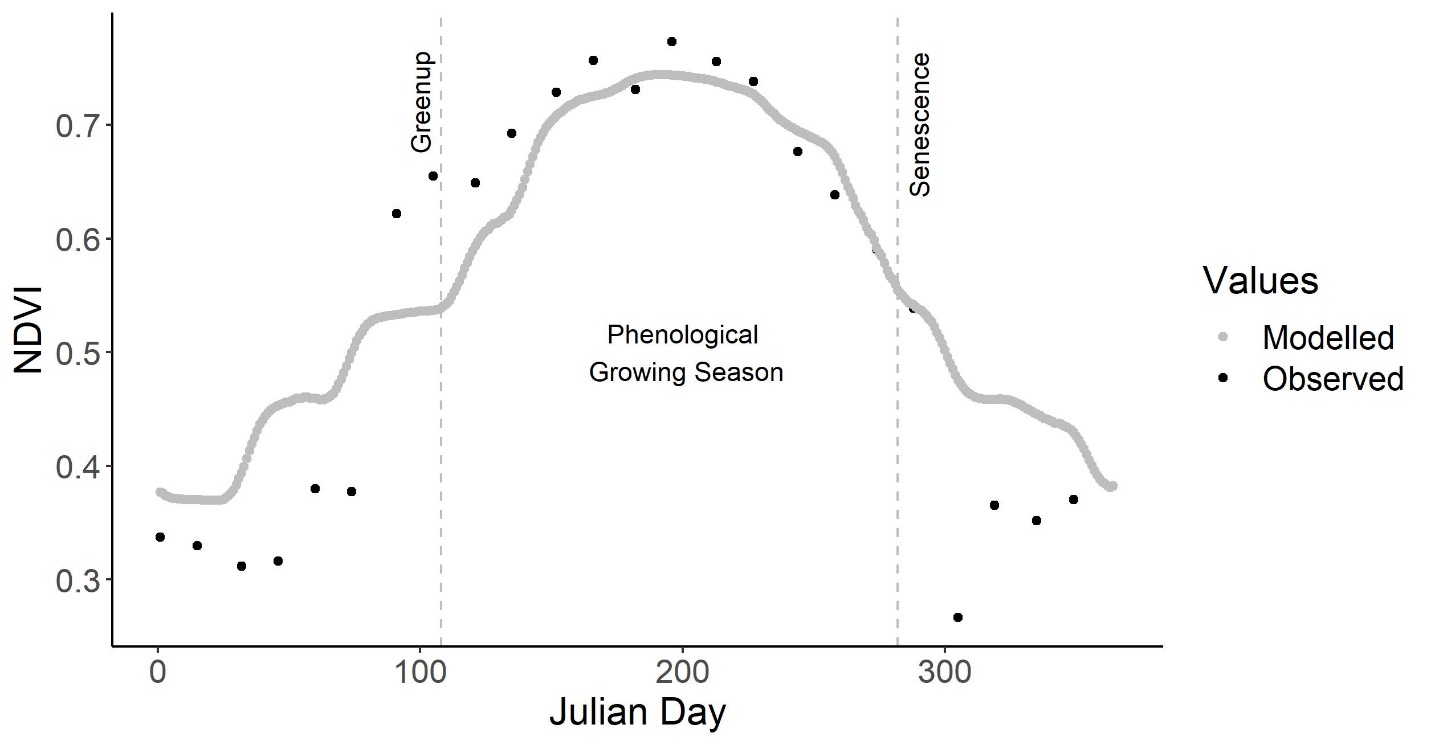


Figure S2.2: Delineation of the phenological growing season within the range of the Cold Lake population of woodland caribou during 1998. A Savitzky-Golay smoothing filter (solid grey line) was fit to the observed NDVI data. The phenological growing season was defined as the period between green-up and senescence.

## Snow Season

We used estimates of snow water equivalents (SWE) from the Daymet data to delineate the yearly snow season in each caribou range. We identified the start and end of the snow season by evaluating for daily changes in the proportion of raster cells (1-km resolution) that were snow-free within each range. To identify the start of the snow season, we used data from 1 October–31 December. The first day of October was used as a starting point to exclude snow storms in September that quickly covered the landscape then subsequently melted (i.e., we wanted to identify the period of continuous snow cover). We used the ‘uik’ function from the ‘inflection’ package (6) to identify the inflection point in the time-series of SWE data when a given caribou range transitioned to none of its cells being snow-free (Fig. S2.3). We applied a similar approach to identify the end of the snow season, using data from 1 January–30 June and looking for the inflection point when most or all of the cells were no longer snow covered.


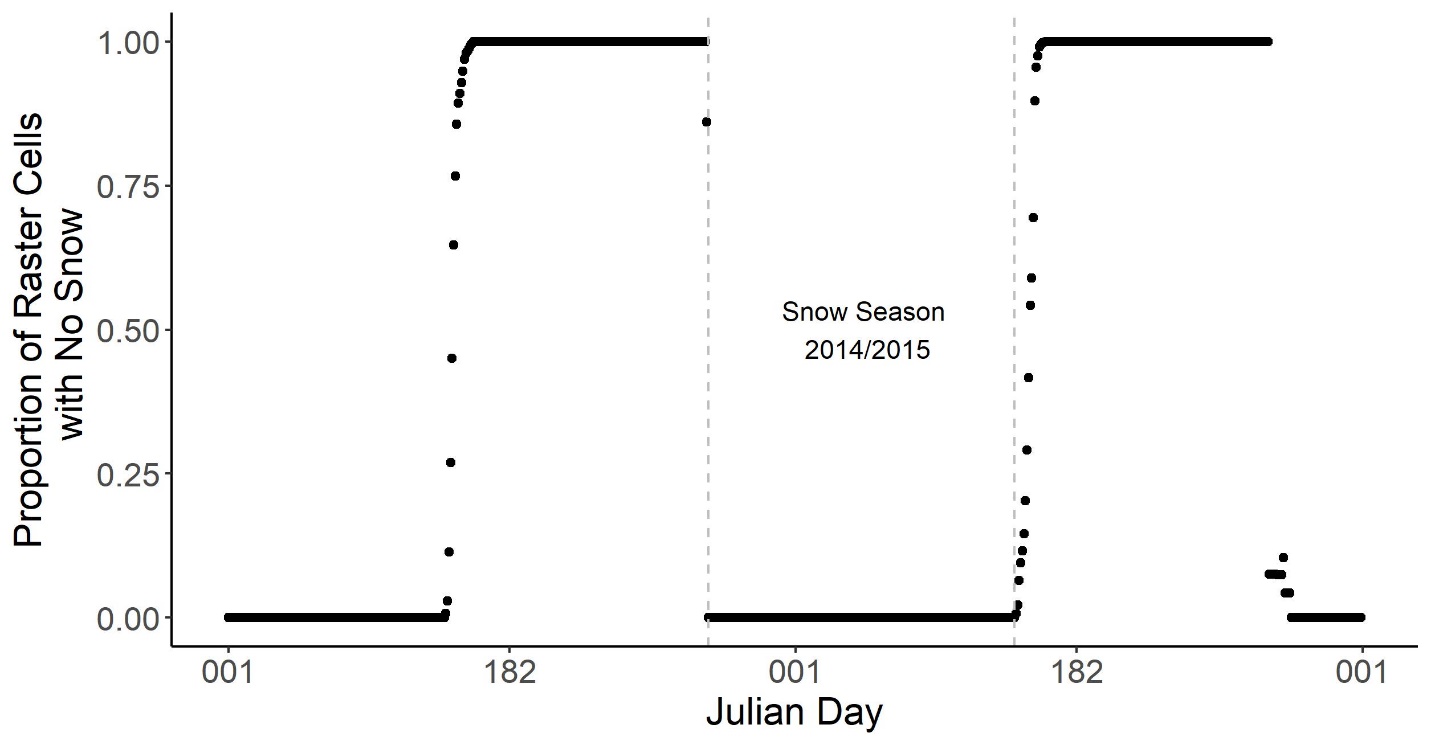


Figure S2.3: Delineation of the snow season within the range of the Yates population of woodland caribou during 2014–2015. The snow season was defined as the period when most or all the raster cells (1-km resolution) were covered by snow within a given caribou range.

*Literature Cited*:

1. Thornton PE, Thornton MM, Mayer BW, Wei R, Devarakonda R, Vose RS, et al. Daymet: Daily surface weather data on a 1-km grid for North America, version 3 [Internet]. Oak Ridge, TN: ORNL DAAC; 2016 [cited 2018 Sep 29]. Available from: https://doi.org/10.3334/ORNLDAAC/1328

2. Liu X, Yin Z-Y, Shao X, Qin N. Temporal trends and variability of daily maximum and minimum, extreme temperature events, and growing season length over the eastern and central Tibetan Plateau during 1961–2003. J Geophys Res. 2006 Oct 12;111(D19):D19109.

3. Lange M, Doktor D. phenex: Auxiliary functions for phenological data analysis. 2017. (R package).

4. Viovy N, Arino O, Belward AS. The Best Index Slope Extraction (BISE): A method for reducing noise in NDVI time-series. Int J Remote Sens. 1992 May;13(8):1585–90.

5. Chen J, Jonsson P, Tamura M, Gu Z, Matsushita B, Eklundh L. A simple method for reconstructing a high-quality NDVI time-series data set based on the Savitzky-Golay filter. Remote Sens Environ. 2004;91(3–4):332–44.

6. Christopoulos DT. inflection: Finds the inflection point of a curve [Internet]. 2019. (R package). Available from: https://CRAN.R-project.org/package=inflection
