# Supplementary material for: Demographic responses of a threatened, low-density ungulate to annual variation in meteorological and phenological conditions
Source: PLoS One. 2021 Oct 8;16(10):e0258136. doi: 10.1371/journal.pone.0258136 (PMC8500449; doi:10.1371/journal.pone.0258136)
Supplement: S3 Appendix — (DOCX) [file pone.0258136.s003.docx]

# S3 Appendix: Diagnostic Testing of Demographic Models

We assessed how annual variation in meteorological and phenological conditions influenced caribou demography using a suite of generalized linear mixed-effects models (GLMMs). Within our modelling framework, we identified two potential sources of bias that could impact inferences from model outputs. The first was potential overdispersion in the calf: adult female (CAF) demographic models. The second was spatial autocorrelation, given the apparent clustering of caribou ranges within Alberta, British Columbia, and the Northwest Territories (see Fig 1 in the main text). Here, we outline the procedures we used to assess for these potential biases and the modelling decisions we made to address these issues if present. We also show procedures for assessing goodness-of-fit for our final CAF and adult female survival (AFS) models.

## Assessing for Overdispersion in Calf: Adult Female Demographic Models

GLMMs with CAF ratios as the response were fit using a binomial distribution and a logit link. We used this specification because twinning is extremely rare in woodland caribou and thus CAF ratios always fall within the 0–1 interval. Ecological models using binomial data are commonly affected by overdispersion, which can bias parameter estimates and standard errors (1). We assessed our CAF models for overdispersion using the ‘DHARMa’ package (2) in R. Below, we show this procedure using data from the meteorologically-defined growing season.

*#Fit mixed-effects model to the data using the package ‘glmmTMB’. This model is assessing the response of CAF to the first principal component ("GS.pc1") during the monitoring year.*

mod1 <- **glmmTMB**((Calves**/**Cows) **~** GS.pc1 **+** trend **+** (1**|**Year) **+** (GS.pc1 **+** trend**|**Herd), family=binomial, weights = Cows, data = grow.dat)

*#Get simulated model residuals*
sim <-**simulateResiduals**(mod1)

*#Assess for overdispersion*
testDispersion(sim)


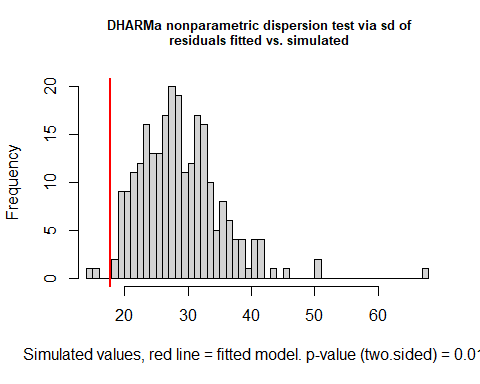


##
## DHARMa nonparametric dispersion test via sd of residuals fitted vs.
## simulated
##
## data: simulationOutput
## ratioObsSim = 0.61991, p-value = 0.016
## alternative hypothesis: two.sided

The above test indicates significant overdispersion in the model. There are two primary ways to account for overdispersion. One is to refit the model using a beta-binomial distribution. The other is to add an observation-level random effect (OLRE) to the model (Harrison 2015). For the first method, there is currently only one package in R that can fit mixed-effects models with a beta-binomial distribution (package ‘glmmTMB’; Brooks et al., 2017). We refit the CAF demographic models using this approach but many models failed to converge. The OLRE approach, in contrast, was much more stable. Although Harrison (2015) proposed the ORLE approach to account for overdispersion in binomial models, he cautioned that the approach did not perform well in all situations and suggested that researchers verify the robustness of model outputs. In the following section, we use data from the phenologically-derived growing season and compare parameter estimates derived from a ‘base’ model with no accounting for overdispersion, a model fit with a beta-binomial distribution, and a model with an OLRE included.

*#This model is assessing the response of CAF to the first principal component ("PH.pc1") during the monitoring year.*
*#Fit 'base' model*
base.mod <- **glmer**((Calves**/**Cows) **~** PH.pc1 **+** trend **+** (1**|**Year) **+** (PH.pc1 **+** trend**|**Herd), family=binomial, weights = Cows, data = ph.dat)
base.df <- **as.data.frame**(**coef**(**summary**(base.mod)))
base.df <- **rownames_to_column**(base.df, "Variable")
base.df <- base.df **%>%** **mutate_at**(**vars**("Estimate", "Std. Error", "z value", "Pr(>|z|)"), **~round**(., 2))
base.df**$**AIC <- **AIC**(base.mod)
base.df**$**model <- "binomial"

*#Fit beta-binomial model*
bb.mod <- **glmmTMB**((Calves**/**Cows) **~** PH.pc1 **+** trend **+** (1**|**Year) **+** (PH.pc1 **+** trend**|**Herd), family=betabinomial, weights = Cows, data = ph.dat)
bb.df <- **as.data.frame**(**summary**(bb.mod)**$**coefficients**$**cond)
bb.df <- **rownames_to_column**(bb.df, "Variable")
bb.df <- bb.df **%>%** **mutate_at**(**vars**("Estimate", "Std. Error", "z value", "Pr(>|z|)"), **~round**(., 2))
bb.df**$**AIC <- **AIC**(bb.mod)
bb.df**$**model <- "betabinomial"

*#Fit OLRE model*
olre.mod <- **glmer**((Calves**/**Cows) **~** PH.pc1 **+** trend **+** (1**|**Year) **+** (1**|**olre) **+** (PH.pc1 **+** trend**|**Herd), family=binomial, weights = Cows, data = ph.dat)
olre.df <- **as.data.frame**(**coef**(**summary**(olre.mod)))
olre.df <- **rownames_to_column**(olre.df, "Variable")
olre.df <- olre.df **%>%** **mutate_at**(**vars**("Estimate", "Std. Error", "z value", "Pr(>|z|)"), **~round**(., 2))
olre.df**$**AIC <- **AIC**(olre.mod)
olre.df**$**model <- "olre"

*#Compare results*
results <- **rbind.data.frame**(base.df[2,], bb.df[2,], olre.df[2,])
results

## Variable Estimate Std. Error z value Pr(>|z|) AIC model
## 2 PH.pc1 -0.05 0.04 -1.27 0.21 1593.944 binomial
## 21 PH.pc1 -0.02 0.04 -0.43 0.67 1451.107 betabinomial
## 22 PH.pc1 -0.02 0.04 -0.50 0.61 1458.644 olre

The above results show that parameter estimates between the beta-binomial model and the OLRE model are comparable and both produce standard errors that are larger (i.e., more conservative) than the ‘base’ model. Because the OLRE approach was generally more stable than fitting models with a beta-binomial distribution, we used the OLRE approach for all CAF demographic models.

## Assessing Spatial Autocorrelation

We also assessed demographic models for potential impacts from spatial autocorrelation because of the apparent spatial clustering of caribou ranges. As a first step, we inspected variograms constructed from each model’s residuals. Variograms were estimated using the ‘variogram’ function from the R package ‘gstat’ (4). Below, we show a variogram constructed from the residuals of a model linking the response of CAF to the first principle component characterizing phenological variation during the monitoring year.

*#Fit model. This model is evaluating the response of CAF to the first principal component characterizing phenological variation ("PH.pc1") during the monitoring year.*
sp.mod1 <- **glmer**(**cbind**(Calves, Cows**-**Calves) **~** PH.pc1 **+** trend **+** (1**|**Year) **+** (1**|**olre) **+** (PH.pc1 **+** trend**|**Herd), family=binomial, data = ph.dat)

*#Get model residuals*
ph.dat**$**resmod <- **residuals**(sp.mod1, type = "pearson")

*#Transform to Spatial Points Dataframe*
**coordinates**(ph.dat) = **~** POINT_X **+** POINT_Y

*#Fit variogram. Assess correlation in four directions*
**plot**(gstat**::variogram**(resmod **~**1, data = ph.dat, alpha=**c**(0,90,180, 270)), xlab=**list**(label="Distance (m)"), ylab = **list**(label="Semivariance"))


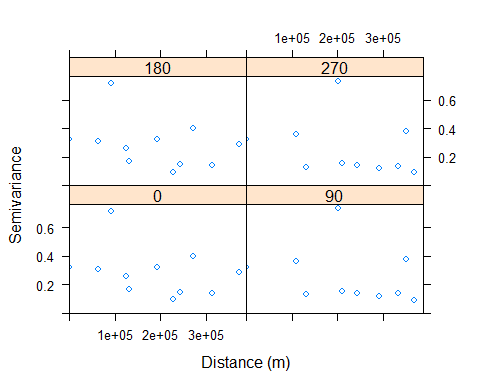


The above variogram does not suggest much influence from spatial autocorrelation. Nevertheless, as a second step, we compared models fit with and without a spatial covariance structure. In R, there are two primary packages for dealing with spatial autocorrelation in GLMMs, though only one is appropriate for our data. In our study design, spatial correlation would only occur among our random grouping factors (i.e., among caribou herds). The repeated measures within each herd do not have a varying spatial component. Of the two R packages, ‘spaMM’ (5) is capable of handling correlation among random grouping factors whereas ‘glmmTMB’ (3) is more suited for correlation occurring within groups. Below, we fit models using the ‘fitme’ function from ‘spaMM’, one with a Matern spatial covariance structure specified and one without. We compared model performance using Akaike’s Information Criterion (AIC). Note that ‘spaMM’ currently does not support >1 random slope within GLMMs.

*#Fit model with Matern spatial covariance*
mat.mod <- **fitme**(**cbind**(Calves, Cows**-**Calves) **~** GS.pc1 **+** trend **+** (1**|**Year) **+** **Matern**(GS.pc1 **+** 0**|**POINT_X **+** POINT_Y), family=binomial, data = grow.dat)

*#Fit base model with no spatial covariance structure*
nospace.mod <- **fitme**(**cbind**(Calves, Cows**-**Calves) **~** GS.pc1 **+** trend **+** (1**|**Year) **+** (GS.pc1**|**Herd), family=binomial, data = grow.dat)

**AIC**(mat.mod)

marginal AIC: 2582.476

conditional AIC: 2464.552

dispersion AIC: 2576.476

effective df: 237.617

**AIC**(nospace.mod)

marginal AIC: 2467.3572

conditional AIC: 2339.0098

dispersion AIC: 2461.3572

effective df: 224.4926

mat.mod.beta <- **summary**(mat.mod)

nospace.mod.beta <- **summary**(nospace.mod)

mat.mod.beta**$**beta_table

## Estimate Cond. SE t-value
## (Intercept) -1.51508540 0.135337354 -11.194880
## GS.pc1 -0.26572758 0.085728479 -3.099642
## trend -0.05695478 0.004261309 -13.365560

nospace.mod.beta**$**beta_table

## Estimate Cond. SE t-value
## (Intercept) -1.71155509 0.132239250 -12.942867
## GS.pc1 -0.19001051 0.054368427 -3.494869
## trend -0.01977898 0.009757889 -2.026974

The above results show that the model with the added spatial covariance structure does not perform better than the base model without such structure (base model >100 AIC unit lower than spatially structured model). These results were consistent for other demographic models and we therefore did not include a spatial covariance structure in our modelling framework.

## Assessing Goodness-of-Fit of Demographic Models

We assessed goodness-of-fit of all demographic models by visually assessing residual plots. For CAF models, we used the ‘plotResiduals’ and ‘testQuantiles’ functions in the ‘DHARMa’ package (2) to fit a quantile regression of simulated residuals from the fitted model against the predicted values. The expectation of a good-fitting model is a uniform distribution of the residuals. Note that the models we ultimately fit are not currently supported by ‘DHARMa’ and therefore these assessments should be viewed cautiously (CAF models were fit with the ‘bglmer’ function from package ‘blme’). Moreover, these tests simply flag models as potentially being problematic, but not necessarily unusable, as fixed-effect estimates may be unbiased even in instances where the residuals deviate from expectation (6). In general, CAF models showed relatively uniform distributions of their simulated residuals. Below, we show outputs for two CAF models where 95% confidence interval for the climate-related fixed-effect variable did not overlap zero.

*#Fit CAF models*

*#Thermo growing season, no lag, PC1*
jvr.mod.pc1 <- **bglmer**((Calves**/**Cows) **~** GS.pc1 **+** trend **+** (1**|**Year) **+** (1**|**olre) **+** (GS.pc1**|**Herd), family=binomial, weights = Cows, data = grow.dat)

mod1_res <- **simulateResiduals**(jvr.mod.pc1)
**plotResiduals**(mod1_res)

**testQuantiles**(mod1_res)


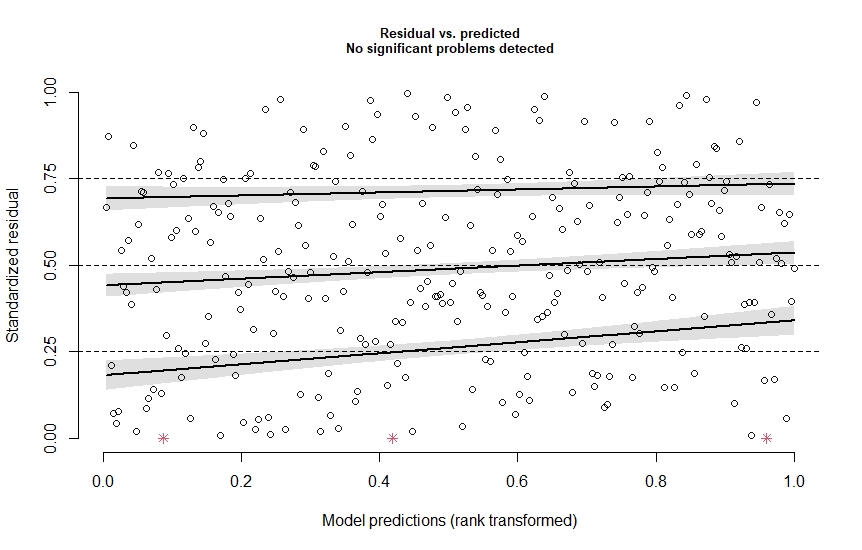


## Test for location of quantiles via qgam

## data: simulationOutput

## p-value = 0.6637

## alternative hypothesis: both

*#Pheno growing season, 1-year lag, PC2*
jvr.ph.lg1.pc2 <- **bglmer**((Calves**/**Cows) **~** PH1.pc2 **+** trend **+** (1**|**Year) **+** (1**|**olre) **+** (PH1.pc2**|**Herd), family=binomial, weights = Cows, data = ph.dat)

mod2_res <- **simulateResiduals**(jvr.mod.pc1)
**plotResiduals**(mod2_res)

**testQuantiles**(mod2_res)


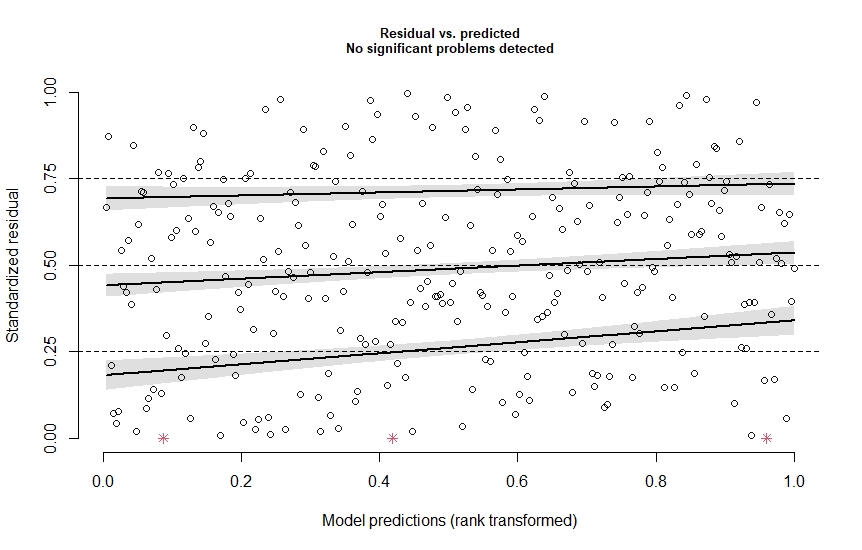


## Test for location of quantiles via qgam

## data: simulationOutput

## p-value = 0.1271

## alternative hypothesis: both

For AFS, mixed-effects models were fit using a beta distribution with a logit link. Diagnostic tests for beta regression models are an active area of research, particularly in a mixed-effects framework, and outputs from the previously used DHARMa functions may not be reliable (2). Because of this uncertainty, we assessed model fit by developing graphs of residuals versus fitted values (code adapted from B. Bolker; <https://stats.stackexchange.com/questions/423274/checking-a-beta-regression-model-via-glmmtmb-with-dharma-package>). Good-fitting models should have mean residual values of zero with no systematic deviations from the mean. In general, most AFS models showed a uniform distribution of their residuals with a few showing slight deviations but no large systematic patterns that could potentially bias the fixed-effect coefficients (6). Below we show graphs from two AFS models where 95% confidence interval for the climate-related fixed-effect variable did not overlap zero.

*#Fit AFS models*

*#Thermo growing season, no lag, PC1*
s.mod.lg1.pc1 <- **glmmTMB**(tS ~ GS1.pc1 + trend + (1|Year) + (GS1.pc1 + trend|Herd), family=beta_family, weights = nS, data = grow.dat)

library(broom.mixed)

mod_afs1 <- **augment**(s.mod.lg1.pc1, data=grow.dat[complete.cases(grow.dat$S),])

afs_p1 <- (**ggplot**(mod_afs1, aes(.fitted,.resid))

+ geom_point()

+ geom_smooth()

)

afs_p1


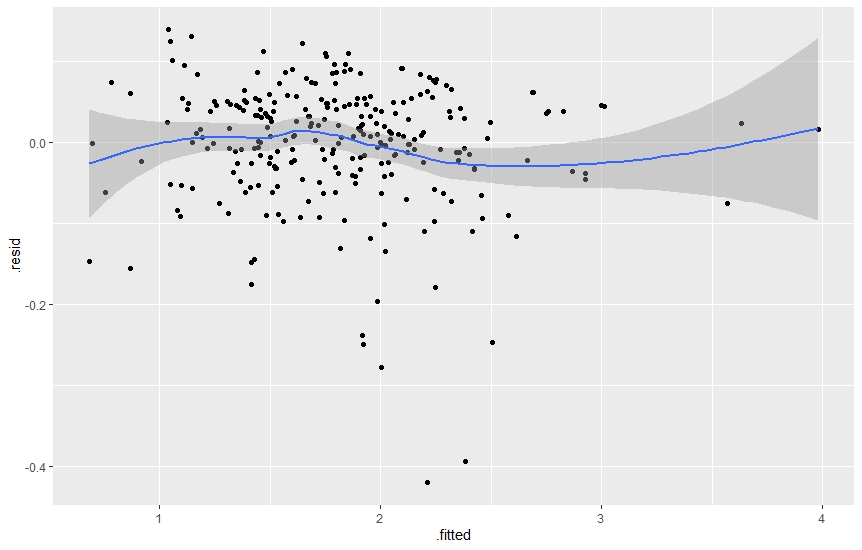


*#Snow season, lag 3, PC2*
s.sn.lg3.pc2 <- **glmmTMB**(tS ~ SS3.pc2 + trend + (1|Year) + (SS3.pc2 + trend|Herd), family=beta_family, weights = nS, data = ss.dat)

mod_afs2 <- **augment**(s.sn.lg3.pc2, data=ss.dat[complete.cases(ss.dat$S),])

afs_p2 <- (**ggplot**(mod_afs2, aes(.fitted,.resid))

+ geom_point()

+ geom_smooth()

)

afs_p2


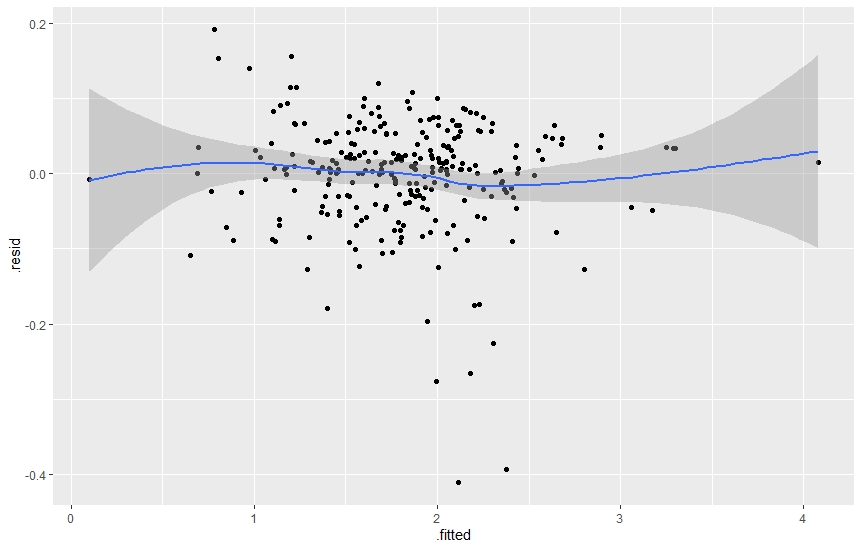


Both plots show evidence of outliers with highly negative values. Inspection of plots depicting the distribution of the random effects in each model shows that these values are likely due to outlying intercept values rather than outlying values of the climate-related variables. This finding suggests that model inferences on climate-related effects are likely minimally influenced by these outlying values.

**lme4:::dotplot.ranef.mer**(ranef(s.mod.lg1.pc1)$cond)


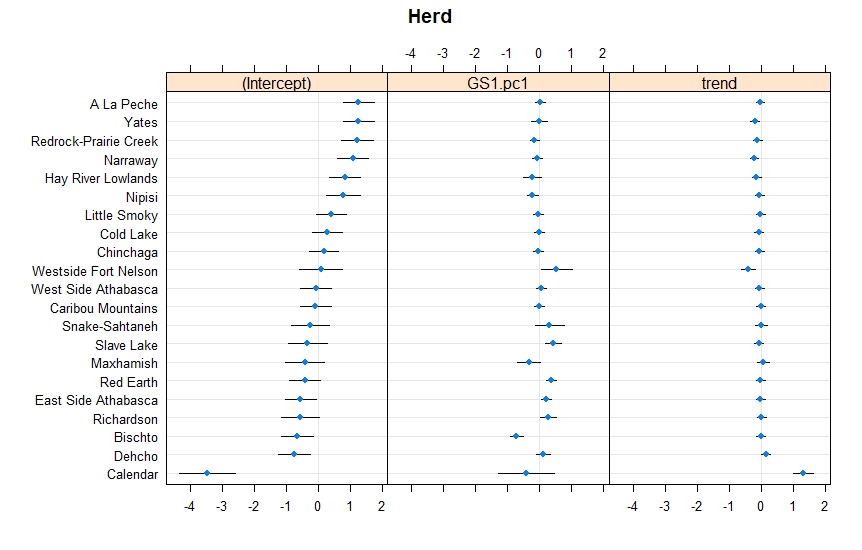


**lme4:::dotplot.ranef.mer**(ranef(s.sn.lg3.pc2)$cond)


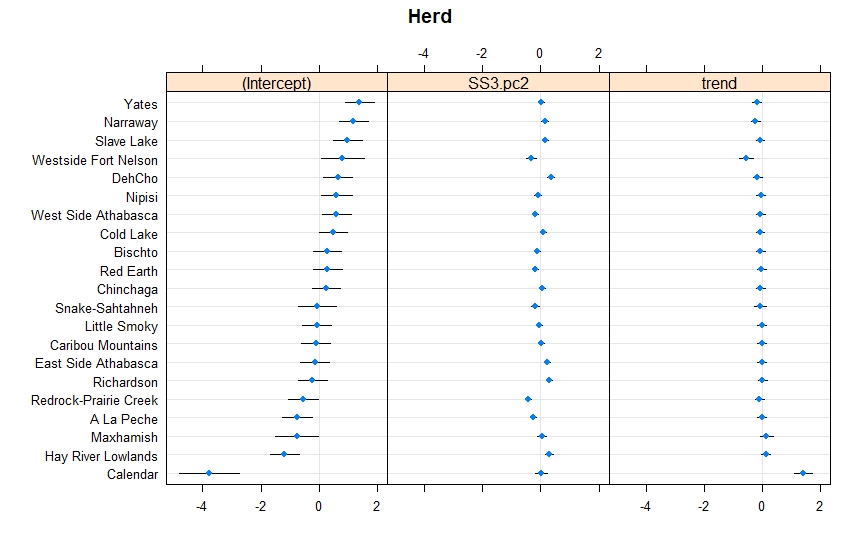


Literature Cited:

1. Harrison XA. A comparison of observation-level random effect and Beta-Binomial models for modelling overdispersion in Binomial data in ecology & evolution. PeerJ. 2015 Jul 21;3:e1114.

2. Hartig F. DHARMa: Residual diagnostics for hierarchical (multi-level / mixed) regression models [Internet]. 2020. (R package). Available from: https://CRAN.R-project.org/package=DHARMa

3. Brooks ME, Kristensen K, van Benthem KJ, Magnusson A, Berg CW, Nielsen A, et al. glmmTMB balances speed and flexibility among packages for zero-inflated generalized linear mixed modelling. R J. 2017;9(2):378–400.

4. Pebesma EJ. Multivariable geostatistics in S: the gstat package. Comput Geosci. 2004;30:683–91.

5. Rousset F, Ferdy J-B. Testing environmental and genetic effects in the presence of spatial autocorrelation. Ecography. 2014;37(8):781–90.

1. Schielzeth H, Dingemanse NJ, Nakagawa S, Westneat DF, Allegue H, Teplitsky C, et al. Robustness of linear mixed‐effects models to violations of distributional assumptions. Methods Ecol Evol. 2020 Sep;11(9):1141–52.
