# Supplementary material for: Demographic responses of a threatened, low-density ungulate to annual variation in meteorological and phenological conditions
Source: PLoS One. 2021 Oct 8;16(10):e0258136. doi: 10.1371/journal.pone.0258136 (PMC8500449; doi:10.1371/journal.pone.0258136)
Supplement: S4 Appendix — (DOCX) [file pone.0258136.s004.docx]

**S4 Appendix: R Packages**

All analyses were performed in R, version 3.6.3 (1), using the packages ‘nlme’ to estimate time-series linear mixed-effects models (2), ‘blme’ to estimate CAF GLMMs (3), ‘glmmTMB’ (4) to estimate AFS GLMMs and ‘MuMIn’ to estimate R^2^ for mixed-effects models (5; note: R^2^ for GLMMs with beta distributions were estimated using code modified from (6)).

*Literature Cited*:

1. R Core Team. R: A language and environment for statistical computing [Internet]. Vienna, Austria: R Foundation for Statistical Computing; Available from: https://www.R-project.org

2. Pinheiro J, Bates D, Debroy S, Sarkar D, R Core Team. nlme: Linear and non-linear mixed effects models [Internet]. 2020. (R package). Available from: https://CRAN.R-project.org/package=nlme

3. Chung Y, Rabe-Hesketh S, Dorie V, Gelman A, Liu J. A nondegenerate penalized likelihood estimator for variance parameters in multilevel models. Psychometrika. 2013;78(4):685–709.

4. Brooks ME, Kristensen K, van Benthem KJ, Magnusson A, Berg CW, Nielsen A, et al. glmmTMB balances speed and flexibility among packages for zero-inflated generalized linear mixed modelling. R J. 2017;9(2):378–400.

5. Barton K. MuMIn: Multi-model inference. 2019. (R package).

6. Nakagawa S, Johnson PCD, Schielzeth H. The coefficient of determination R2 and intra-class correlation coefficient from generalized linear mixed-effects models revisited and expanded. J R Soc Interface. 2017;14:20170213.
