# Supplementary material for: Demographic responses of a threatened, low-density ungulate to annual variation in meteorological and phenological conditions
Source: PLoS One. 2021 Oct 8;16(10):e0258136. doi: 10.1371/journal.pone.0258136 (PMC8500449; doi:10.1371/journal.pone.0258136)
Supplement: S5 Appendix — (DOCX) [file pone.0258136.s005.docx]

**S5 Appendix: Expanded Results of Principal Component Analyses**

Principal component analyses (PCAs) were used to summarize the suite of meteorological and phenological variables considered to potentially influence caribou demography within a given season. Here, we provide further details into the relative contribution of each variable to the first two principal components (PCs) derived from the PCAs. These PCs were subsequently used as explanatory variables in generalized linear mixed-effects models.

*Meteorological Growing Season*

For PCAs developed for the meteorological growing season, the first two PCs were primarily influenced by season length, the end date of the season, and the mean maximum and minimum temperatures. The relative contribution of variables to the first PC was similar across all time periods (monitoring year and lags up to three years) and >90% of the relative contribution was attributed to five of the eight variables: season length (22–23%, positive loadings), end date (20–21%, positive loadings), start date 18–19%, negative loadings), SD of the minimum temperature (16–17%, positive loadings) and mean minimum temperature (12–14%, positive loadings). Based on these variable contributions, the first PCs generally described longer growing seasons with warmer but more variable minimum temperatures. The growing seasons characterized by the second PCs varied slightly depending on the time period. For the monitoring year and two- and three-year lags, the second PCs described cooler and wetter growing seasons with >76% of the relative variable contribution attributed to the mean maximum temperature (38%, negative loading), the mean minimum temperature (13–19%, negative loading) and cumulative precipitation (21–30%, positive loading). The second PC for one-year lags had similar contributions but different directional loadings (mean maximum temperature: 39%, positive loading; mean minimum temperature: 16%, positive loading; cumulative precipitation: 30%, negative loading) and consequently described growing seasons that were warmer and drier.

*Phenological Growing Season*

Among the phenological variables considered, growing season length, the date of senescence in the fall, and iNDVI had the highest relative contribution to the first two PCs of period-specific PCAs. Across all time periods, the first PCs described longer, more productive growing seasons as >60% of the relative contribution to these components was attributed to season length (32–39%, positive loadings) and iNVDI (24–28%, positive loadings). Seasonal descriptions of the second PCs varied by time period. For the monitoring year and two- and three-year lags, the second PCs described growing seasons that were less productive, had later senescence and a delayed peak of NDVI values. At least 82% of the relative contribution to these second PCs was attributed to the date of maximum NDVI value (30–33%, positive loadings), the maximum NDVI value (23–30%, negative loadings), and the date of senescence (20–29%, positive loadings). For one year lags, the second PC described growing seasons that were productive but productivity peaked earlier and senescence occurred earlier. The relative contribution of variables to this component was similar to the other second PCs (combined contribution of senescence (negative loading), maximum NDVI date (negative loading) and maximum NDVI value (positive loading) = 83%).

*Snow Season*

PCAs developed for the snow season had a relatively even contribution from the input variables (*n* = 12) into their first two PCs. Cumulative SWE had the highest relative contribution at 11% but seven other variables had contributions >9% (March snow, mean and SD of SWE, mean and SD of minimum temperature, mean and SD of maximum temperature). For the monitoring year and first two lags, the first PCs generally described shorter snow seasons with low snow accumulations as >84% of the contribution to these PCS were attributed to cumulative SWE (16–18%, negative loading), March snow (16–17%, negative loading), mean SWE (15–16%, negative loading), SD SWE (14–15%, negative loading), season length (12%, negative loading), and season end (11–12%, negative loading). The first PC using three-year lag data described snow seasons that were the opposite—longer with more snow—as this PC had the same suite of variables providing similar contributions but all loadings were positive. Second PCs primarily characterized snow seasons in terms of temperature. Across all time periods, the second PCs generally described colder seasons with increased temperature variability as >81% of the relative contributions to these PCs were attributed to the mean (23–24%, negative loading) and SD (16–19%, positive loading) of the maximum temperature, and the mean (23%, negative loading) and SD (18–20%, positive loading) of the minimum temperature. Of note, the fifth-ranked variable in terms of relative contribution was the number of freeze-events (positive loading), but its relative contribution was <7.5%.
