# Supplementary material for: Demographic responses of a threatened, low-density ungulate to annual variation in meteorological and phenological conditions
Source: PLoS One. 2021 Oct 8;16(10):e0258136. doi: 10.1371/journal.pone.0258136 (PMC8500449; doi:10.1371/journal.pone.0258136)
Supplement: S6 Appendix — (DOCX) [file pone.0258136.s006.docx]

# S6 Appendix: Correlations Among Meteorlogical and Phenological Variables

To assess the demographic response of woodland caribou to climatic variation, we used to two different approaches to model the growing season. One approach used meteorologically-derived variables including the last day of spring frost, the first day of fall frost and growing season length (the interval between spring frost and fall frost). The other approach used variables indexing changes in plant phenology. These included estimates of the date of green-up in the spring, the date of plant senescence in the fall, and growing season length (the interval between green-up and senescence). Here, we assessed the strength of correlation among these variables using Pearson’s correlation coefficient (Fig S6.1). Correlations between meteorological and phenological variables did not exceed |0.16|, suggesting that the two seasonal approaches were capturing different seasonal characteristics. These differences also translated to only weak correlations among the principal components used to define the two types of growing seasons. For example, Pearson’s correlation coefficients among the first two principal components using one-year lagged data were ≤0.26 (Fig S6.2).


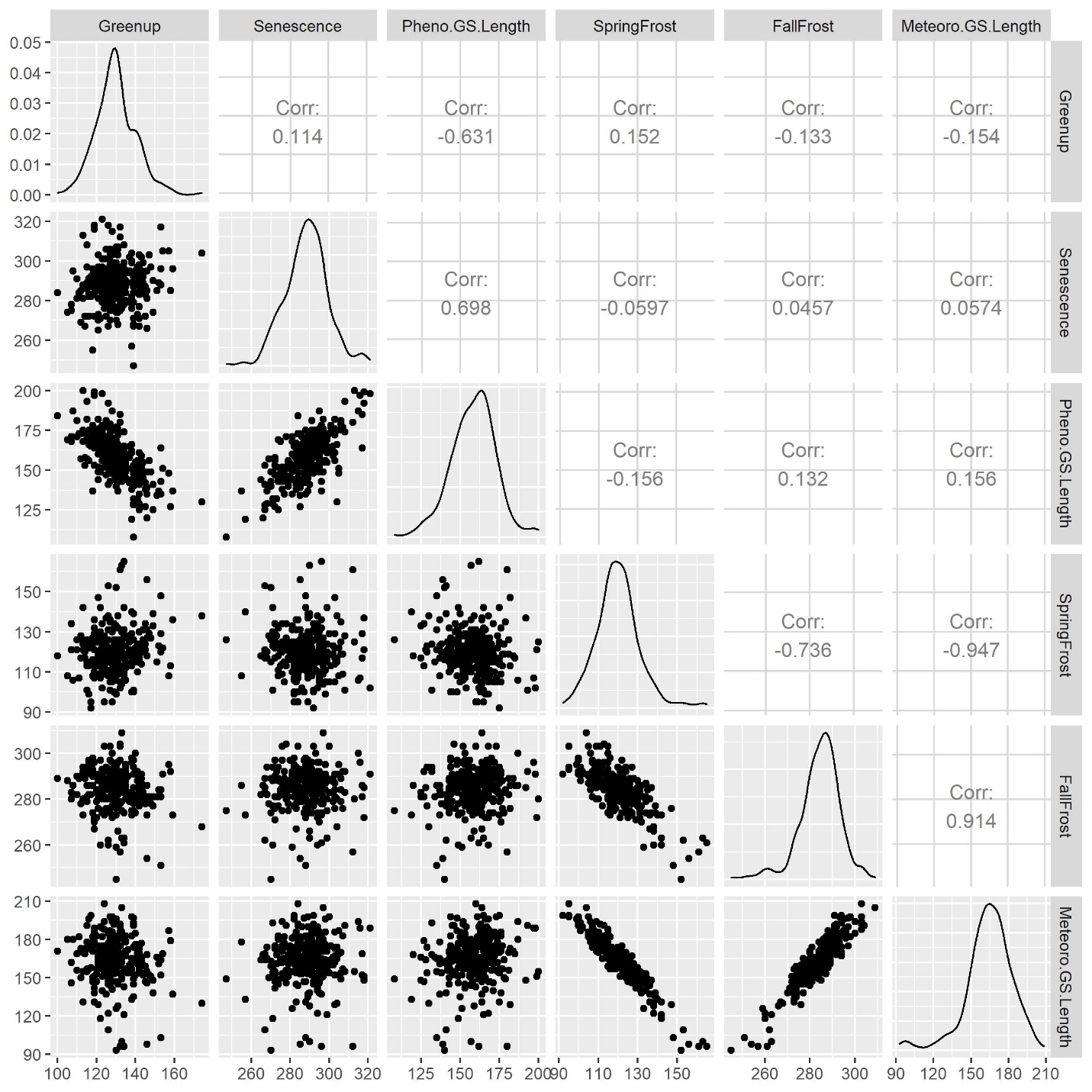


Figure S6.1: Relationships among meteorological and phenological variables used to model the growing season within the ranges of 21 populations of woodland caribou. “Corr” indicates the strength of the correlation (measured by Pearson’s correlation coefficient) between the column and row variables. Line graphs on the diagonal show the probability density function of each variable. Note that the x- and y-axes vary among graph facets.


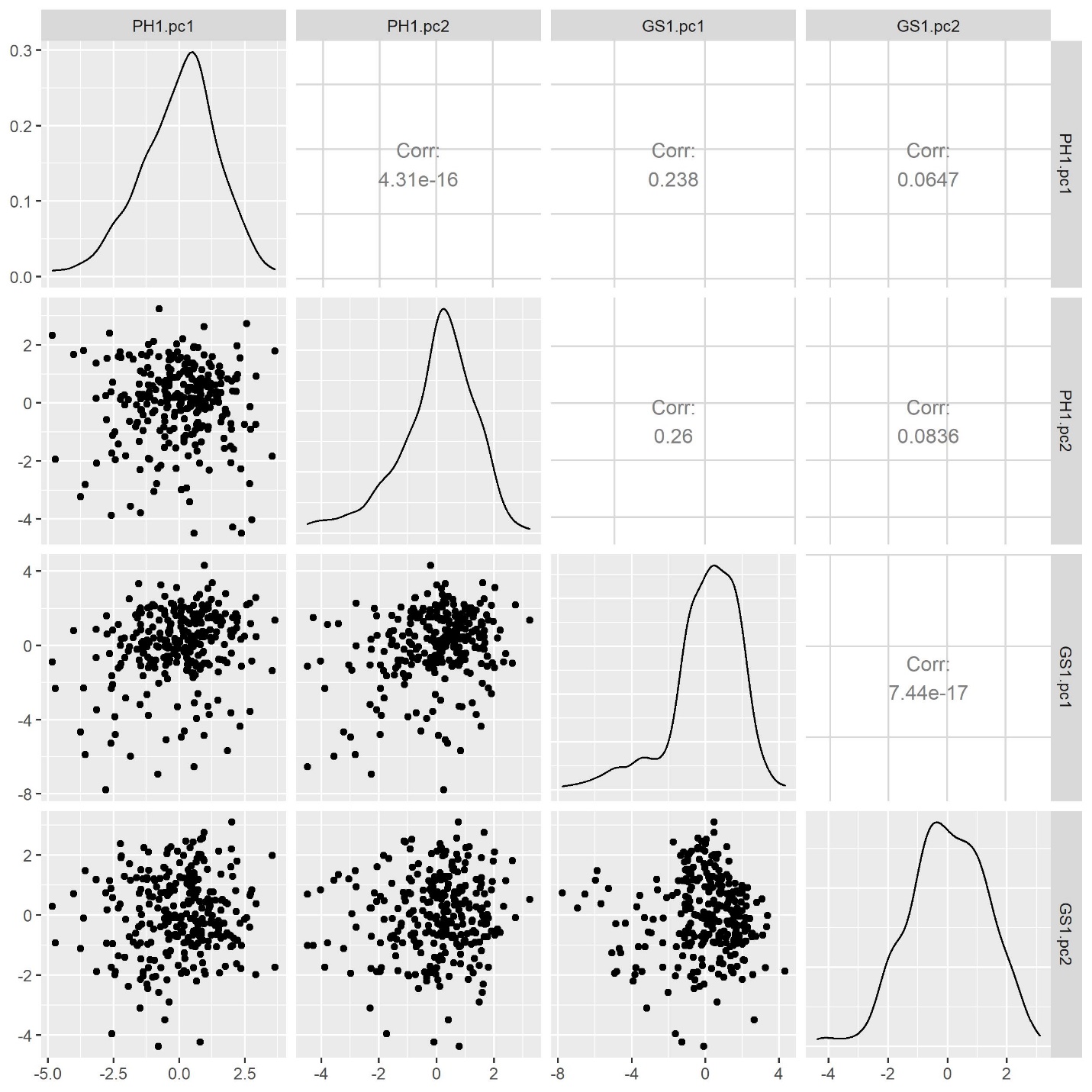


Figure S6.2: Relationships among the first two principal components from the meteorological growing season (GS1.pc1 and GS1.pc2) and the phenological growing season (PH1.pc1 and PH1.pc2) using one-year lagged data. Principal component analyses were conducted using meteorological variables and, separately, phenological variables to model meteorologically-defined growing seasons and phenologically-defined growing seasons within the ranges of 21 populations of woodland caribou. “Corr” indicates the strength of the correlation (measured by Pearson’s correlation coefficient) between the column and row variables. Line graphs on the diagonal show the probability density function of each variable. Note that the x- and y-axes vary among graph facets.
